# Stable and efficient transfer-printing including repair using a GaN-based microscale light-emitting diode array for deformable displays

**DOI:** 10.1038/s41598-019-47449-1

**Published:** 2019-08-09

**Authors:** Jun-Beom Park, Keon Hwa Lee, Sang Hoon Han, Tae Hun Chung, Moon Kyu Kwak, Hokyun Rho, Tak Jeong, Jun-Seok Ha

**Affiliations:** 1 Korea Photonics Technology Institute, Photonic Device Research Center, 124, Cheomdanventure-ro, Buk-gu, Gwangju 61007 Republic of Korea; 2 Chonnam National University, Optoelectronics Convergence Research Center, 77, Yongbong-ro, Buk-gu, Gwangju 61186 Republic of Korea; 3 Kyungpook National University, Mechanical Engineering, 80, Daehak-ro, Buk-gu, Daegu, 41566 Republic of Korea

**Keywords:** Electrical and electronic engineering, Inorganic LEDs

## Abstract

GaN-based microscale light-emitting diodes (μLEDs) are reported for assembly into deformable displays and repair systems. A stamp-imprinting method that enables large area assembly without spatial limitation is involved in the system, and a selective pick-up method is presented that includes a method for removing detected defective chips through micro-pulsed laser scanning. The photosensitive functional material, which is an accepted layer for the stable imprinting of chips, is determined by controlling the adhesion. In addition, selective pick-up and adhesion-controlled functional materials allow the implementation of defect-free displays through two pick-and-place cycles. Displays and related systems fabricated with this method can offer interesting optical and electrical properties.

## Introduction

Recently, ultra-high-resolution displays have attracted attention because of the development of applications such as smart phones, glass monitors, and virtual-reality devices. These ultra close-up monitors require a high pixel per inch (PPI) because the pixels are seen to be relatively larger at a visual distance of 2 cm–3 cm^1,2^. In line with this trend, researchers are focusing on the development of microscale light sources. Currently, there are various types of displays depending on the driving method and light source, such as liquid crystal displays (LCDs), plasma display panels (PDPs), organic light emitting diode (OLED) displays, and inorganic light emitting diode (ILED) displays. LCDs are difficult to apply to ultra-high-resolution displays because of the minimum space needed for the liquid crystal and the blocks that divide the pixels^3,4^. PDPs are also not suitable because of the block dividing pixels and the burn-in phenomenon. OLED displays are based on organic materials with strong aggregation characteristics and thus have limitations in forming ultra-high-resolution displays^5,6^. On the other hand, ILED displays are advantageous for manufacturing ultra-high-resolution displays because the technology is capable of pixel-diving in microscale units and is based on photolithography through growing or depositing^7,8^. However, it is difficult to fabricate microscale LEDs (μLEDs) in a display form, because of problems such as low optical efficiency, low crystalline GaN, chip packaging, and chip defect analysis as the size of the LEDs is reduced in the microscale^9,10^. The most urgent among the many problems is pick-and-place from the LED wafer to the board substrate. In the case of conventional large LEDs, one chip is picked up and placed on a board substrate repeatedly to fabricate a display. However, the smaller size of the LED chips imposes more restrictions on moving single chips. Thus, many methods have been proposed to efficiently transfer μLEDs. A method of transferring an array after temporarily fixing μLED chips using photoresist (PR) or polymethylmethacrylate (PMMA) laminating has been attempted^11,12^. Photolithography-based PR has a pattern restriction, and PMMA requires all the chips to be transferred at once. Therefore, a repair step must be added. A method of fixing and transferring μLED arrays with an anchor pattern was also tried^13^. This approach requires space for the anchors to support the chips, so there is a limit to fabricating a dense array. Instead of vacuum force, electrostatic force can be used to pick up and transfer the chips^14^. The method of using electrostatic force has the risk of interference among densely arranged chips. In addition, there is a method of separating and transferring wafer and μLED chips through wet etching^15^. However, there is a risk that LEDs composed of various materials can be damaged during wet etching.

The stamp-imprinting method can overcome the above-mentioned limits. The imprinting method using stamps has a great advantage in moving large quantities of chips at a time. However, the stamp-imprinting method has a fatal drawback in that defective chips must be transferred with good-quality chips. Therefore, after imprinting the μLED array using a stamp, it is necessary to remove the defective chip and replace the good chip in the empty space where the defective chip was removed. Recently, a method of transferring a good chip beside the defective chip without removing them has been used for manufacturing displays^16^. However, considering manufacture of displays with a higher pixel density, this method is not the ultimate solution because it is difficult to secure space for a spare chip. The solution to this problem is the top priority for manufacturing μLED displays using stamp-imprinting.

In this paper, we propose a precise, stable, and cost-effective pick-and-place method that can be repaired. The transfer is defined in four steps as follows: (i) Analysis of the defective chips in a μLED wafer, (ii) selective pick-up from μLED wafer to stamp, (iii) imprinting of the μLED array to the board substrate using a functional layer capable controlled-adhesive force, and (iv) repair. We can pick up only the good μLED chips, excluding the defective chips, using UV pulsed excimer (UV PE). In addition, a micro-pulsed laser was used to analyze the defective cihps by scanning the μLED wafer^17,18^. The stamp used to transfer the μLED array from the wafer to the board substrate is polydimethylsiloxane (PDMS), which is a dry adhesive silicone rubber^19,20^. These picked-up good chip arrays are then imprinted on a board substrate with a UV-photosensitive thin film. This photosensitive functional layer controls the adhesive force through UV exposure and is completely full-cured when the transfers are completed. To verify the stamp-imprinting method developed in this study, we fabricated a passive-matrix prototype after transferring single chips and a 10 × 10 array to a deformable polyimide (PI) substrate.

## Results

**Figure 1:**
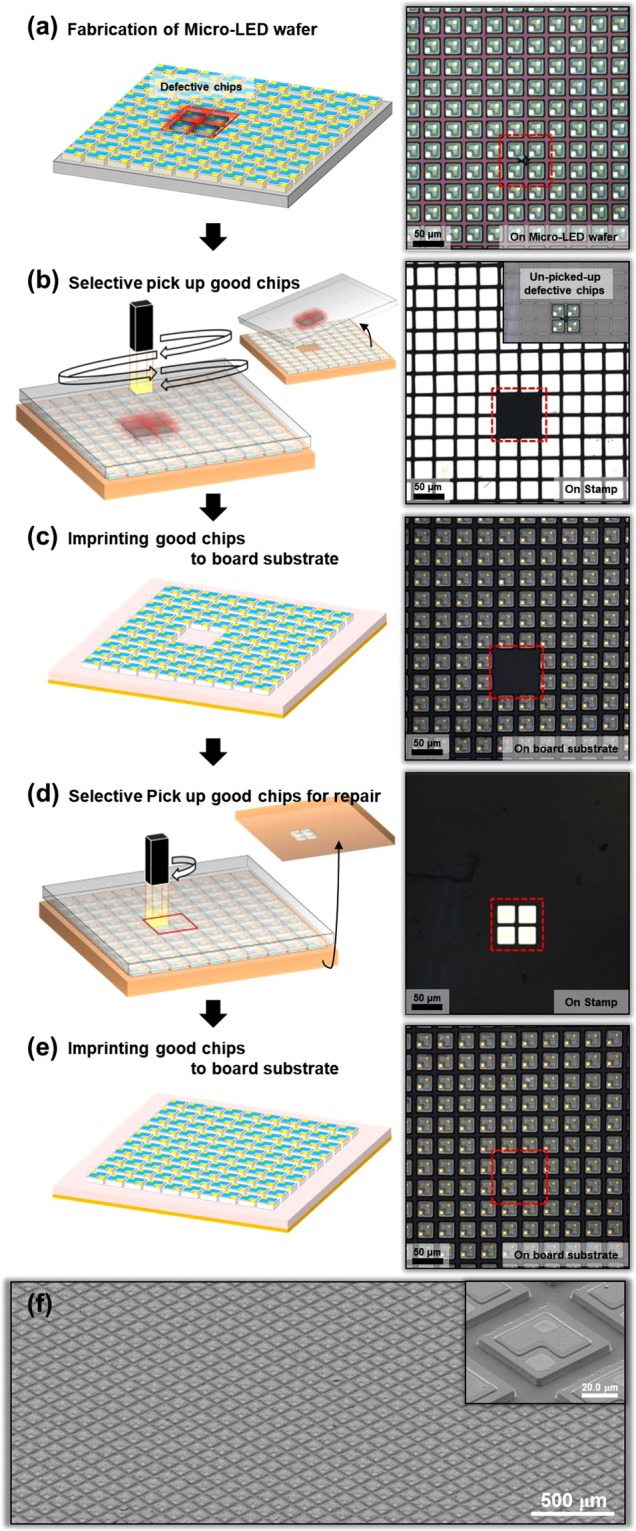
Schematic illustrations and images corresponding to the steps for forming, assembling, and repairing ultrathin μLEDs fabricated from GaN on sapphire substrates: (**a**) μLED wafers including defective chips (left: schematic; right: optical microscopy image). (**b**) Selective pick-up of the array of good chips from the μLED wafer to stamp by UV PE (left: schematic; right: optical microscopy image). (**c**) Imprinting the array of good chips to the PI board substrate that coated the functional layer (left: schematic; right: optical microscopy image). (**d**) Selective pick-up of the array of good chips for repair from μLED wafer to stamp according to the coordinates of empty spaces on the PI board substrate (left: schematic; right: optical microscopy image). (**e**) Align-imprinting the array for repairing the array on the PI board substrate (left: schematic; right: optical microscopy image). (**f**) SEM images of the transferred array on the PI board substrate.

Figure 1 outlines the pick-and-place steps, in a sequence of schematic illustrations, microscopy images, and photographs. Figure 1(a) shows μLED chips on sapphire, etched into square islands (50 × 50 μm^2^) with an L-shaped mesa part (0.5 μm depth), current-spreading layers, and n- and p-pads. Figure 1(b) shows the selective pick-up where only good chips are picked up to stamp, excluding the defective chips caused during μLED fabrication. After the μLED wafer and the stamp are attached, the μLED chip or chips are separated from the wafer by the irradiation of UV PE to the back side of the μLED wafer. The detailed mechanism of pick-up is described in the Supporting Information. The main focus of the work presented here is to irradiate the desired portion of the UV pulse and adjust the irradiation area. Irradiation of the UV pulse at the desired portion means that only good chips are picked up by irradiating only the portion of the good chips and excluding the defective chips. In addition, it means that this method includes a repair step. In addition, the adjustment of the irradiation area means that a single UV pulse can pick up many chips at once, which is a highly useful advantage for large-area transfer (see Supporting Information and Figure S2 for details). The microscopy images of Figure (b) show that defective chips remain on the μLED wafer and only good chips are picked up on the stamp. Next, the array on the stamp is imprinted on the board substrate. At this time, because the arrays are held by the sticky characteristics of the PDMS stamp, the board substrate needs a functional layer having higher adhesive force than the stamp. Furthermore, after all the chips are imprinted to the functional layer, the functional layer must harden so that the chip can be fixed. The imprinted array can be seen in Fig. 1(c). In addition, the repair step is needed because of the empty spaces on the functional layer due to the selective pick-up. After scanning the coordinates of the empty spaces, good chips from the μLED wafer are picked up using the selective UV pulse according to their coordinates. This array for repair is then aligned-imprinted on the array of functional layers. Figure 1(f) shows the scanning electron microscope (SEM) images of the transferred array on the PI board substrate.

**Figure 2:**
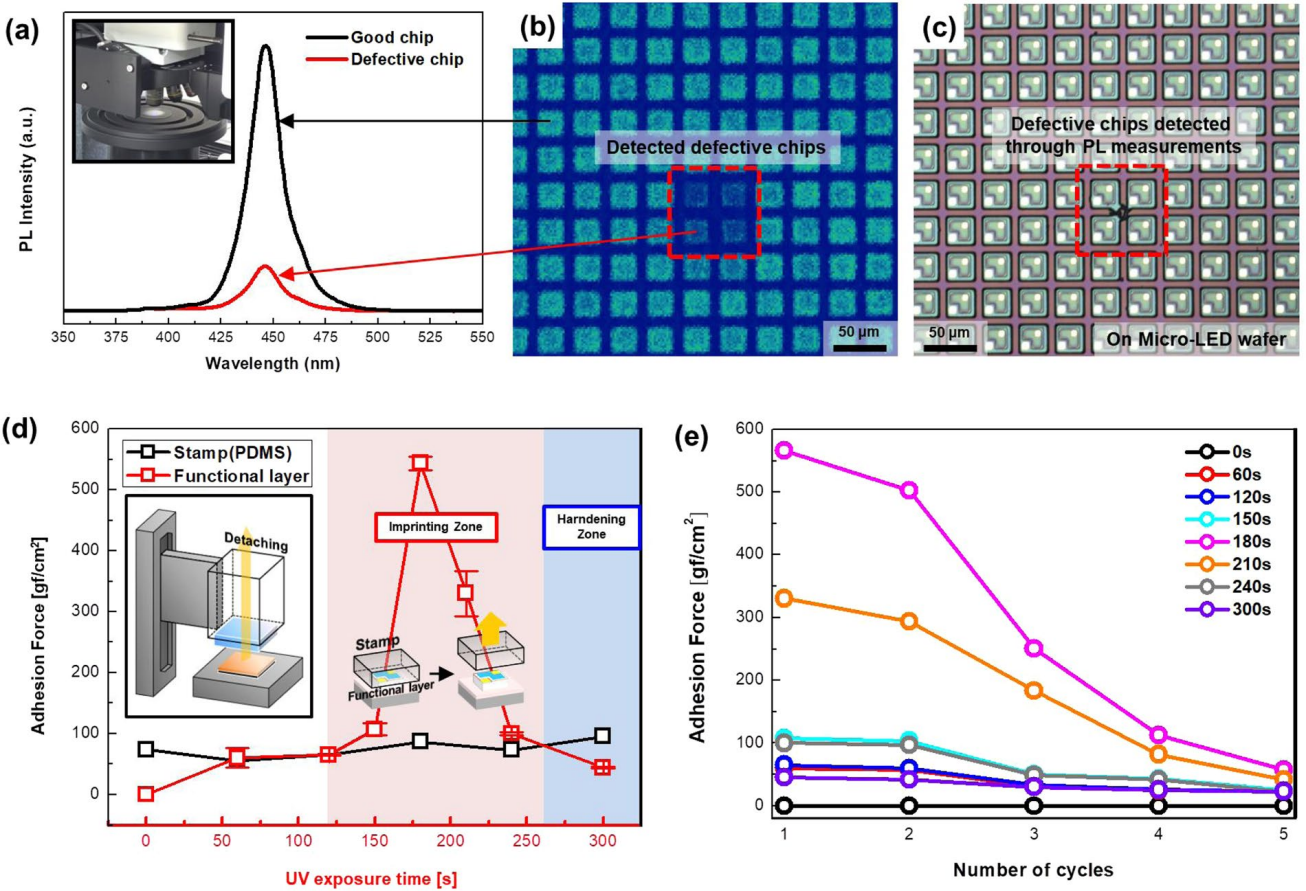
(**a**) PL intensity of good and defective chips detected by micro-pulsed laser (inserted image: Schematic illustrating how to measure adhesion; after a 1.5 × 1.5 cm^2^ GaN piece on sapphire and the functional layer on the PI substrate are attached, the force generated by detaching these samples was measured). (**b**) Mapping image of the detected PL intensity after micro-pulsed laser irradiation. (**c**) Optical microscopy image of defective chips on a real μLED wafer found using a PL intensity map. (**d**) Graph of the adhesion force of the stamp and the functional layer according to UV exposure time. (**e**) Graph of the repeated adhesion force of the functional layer according to UV exposure time (0 s–300 s at intervals of 60 s).

After fabricating the μLEDs, good chips and defective chips should be analyzed to select the chips to be transferred. Because the number of chips fabricated on a wafer is quite large, it takes a long time to probe each chip. In addition, it is difficult to contact the probe because the size of the chip is extremely small. Therefore, we analyzed the μLED chips using a micro-pulsed laser emitting light at 375 nm. The pulse of the micro-pulsed laser is irradiated to the μLED chips and meets multi-quantum wells (MQWs) based on InGaN through thin p-type GaN. At this time, the MQWs having a band gap of 2.7 eV absorb the light energy of the pulse having an energy of 3.3 eV, and recombination of electrons and holes occurs^21^. This recombination leads to emission at 450-nm wavelength, and the remainder that is not used for recombination is converted to thermal energy^22,23^. The photoluminescence (PL) intensity of the re-emitted 450-nm wavelength is detected, and defective chips are determined on the basis of their relatively lower intensity; the low PL intensity is usually found by the leakage current of defective chips. In addition, a laser with a diameter less than 2 μm was used for accurate analysis for the small size of the chip. Figure 2(a–c) show a graph and images of μLED analysis using the micro-pulsed laser. The inserted image of Fig. 2(a) is the micro-PL scan of the μLED wafer. The time required for full-scanning a 2-inch μLED wafer is less than 5 min. Figure 2(b) shows the mapping image of the detected PL intensity after micro-pulsed laser irradiation, in which chips with four low-PL intensities were found. The PL intensity of the good chip and the PL intensity of the defective chip are shown in Fig. 2(a). Using the mapping image, it was confirmed that four chips were unsuitable for the isolation pattern from the real μLED wafer. Failure of the isolation pattern led to leakage current and decreased PL intensity.

Figure 2(c,d) show graphs of the adhesion force of the functional layer. A mercapto-based polymer containing a photo acid generator (PAG) is used as the functional layer. The major components of the functional layer are mercapto-ester and triallyl isocyanurate. The adhesion force graph of the PDMS stamp and functional layer according to UV exposure time are shown in Fig. 2(d). The inserted schematic describes the measurement method of the adhesion force. When the bonded GaN wafer and functional layer on the PI substrate are detached, the sensed force is detected. The illuminance of the UV exposure equipment is 7 lm/m^2^ to improve the adhesion of the functional layer, and the distance between the UV light source and sample is 5 cm. The adhesion force of the functional layer before UV exposure was lower than that of the stamp, but gradually increased from 50 s to 150 s; after 150 s, the adhesion increased suddenly. The sudden increase in adhesion is due to the reaction of the PAG of the functional layer when exposed to UV light, and the adhesion of the functional layer is improved by the coexistence of a fluid polymer and an ester bond reacted by UV exposure^24^. After UV exposure for 180 s, the adhesive force decreases and hardens through the completion of the PAG reaction. Thus, the array can be imprinted to the functional layer exposed from 150 s to 230 s, which is a higher adhesive force than that of the PDMS stamp. The functional layer having an adhesive force of 566 gf/cm^2^ after coating the functional layer on the board substrate and then UV exposure for 180 s at a luminance of 7 lm/m^2^ was used for imprinting. In addition, because a transfer is required again for repair after the first transfer, it is necessary to confirm the repeated adhesion force of the functional layer. The graph of the repeated adhesion force of the functional layer is shown in Fig. 2(e). The adhesion force over 5 cycles was measured to the functional layer UV-exposed up to 300 s by increasing at intervals of 60 s. The adhesion forces of the functional layer exposed for 180 s were 566.2 gf /cm^2^, 502.1 gf/cm^2^, 250.7 gf/cm^2^, 112.7 gf/cm^2^, and 57.8 gf/cm^2^ according to repeated adhesive cycles. These results of higher adhesion forces than this PDMS stamp indicate that transfer for repair is possible. In addition, the functional layer UV-exposed during 0 s, 60 s, 120 s, 150 s, 210 s, 240 s, and 300 s showed a similar tendency of decreasing the adhesion force depending on the number of cycles.

**Figure 3:**
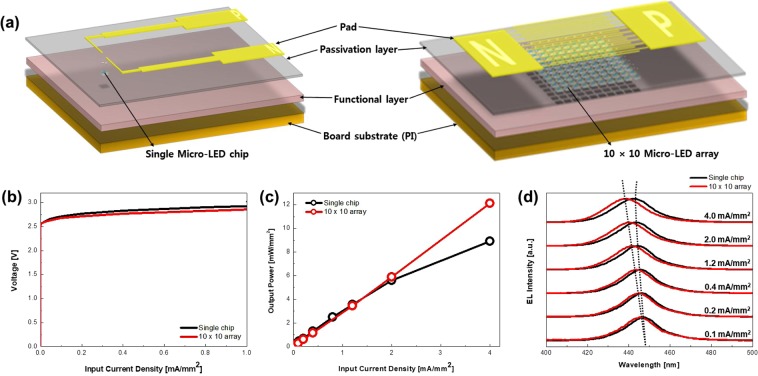
(**a**) Schematic of a single chip and 10 × 10 μLED chips in the passive matrix (PM). (**b**) Voltage-input current density (V-I) characteristics of a single chip and array. (**c**) Output power density-input current density (Po-I) characteristics of a single chip and array (inserted left image: emission of single chip at 0.1 mA, inserted left image: emission of array at 0.1 mA). (**d**) Electroluminescence (EL) spectra of a single chip and array.

Figure 3 shows the detailed experimental results of the array obtained using selective pick-up. Figure 3(a) schematically illustrates the device configuration of single chip and array. The array was composed of 10 × 10 μLED chips in the passive matrix (PM); see Supporting Information and Figure S3 for details. Comparison of the single chip and array (Fig. 3(b,c)) reveals nearly identical forward voltage and output power density at low current density. However, as the input current density increases, the voltage of the single chip increases compared to the characteristics of the array. In addition, the output power density of a single chip becomes lower than that of the array with increasing input current density higher than 2 mA/mm^2^. Figure 3(d) shows the electroluminescence (EL) spectra of a single chip and array, which in turn explains why the single chip has a lower output power density than the array with increasing input current density. The initial value of the dominant wavelength of EL for both devices is the same at 445 nm, and a blue shift is observed in both devices upon a further increase in current density until 1.2 mA/mm^2^, because the electron and hole population increased and led to a band filling effect^25^. However, upon further increase in current density, a different tendency of the wavelength shift is observed in that the red shift of the dominant wavelength occurs only in a single chip owing to the reduced bandgap with junction temperature rise^26^. Unless the thermal effect is not considered, the fabricated array using selective pick-and-place in this study is noteworthy in that the optical and electrical characteristics of the single chip are almost the same at low input current density.

**Figure 4:**
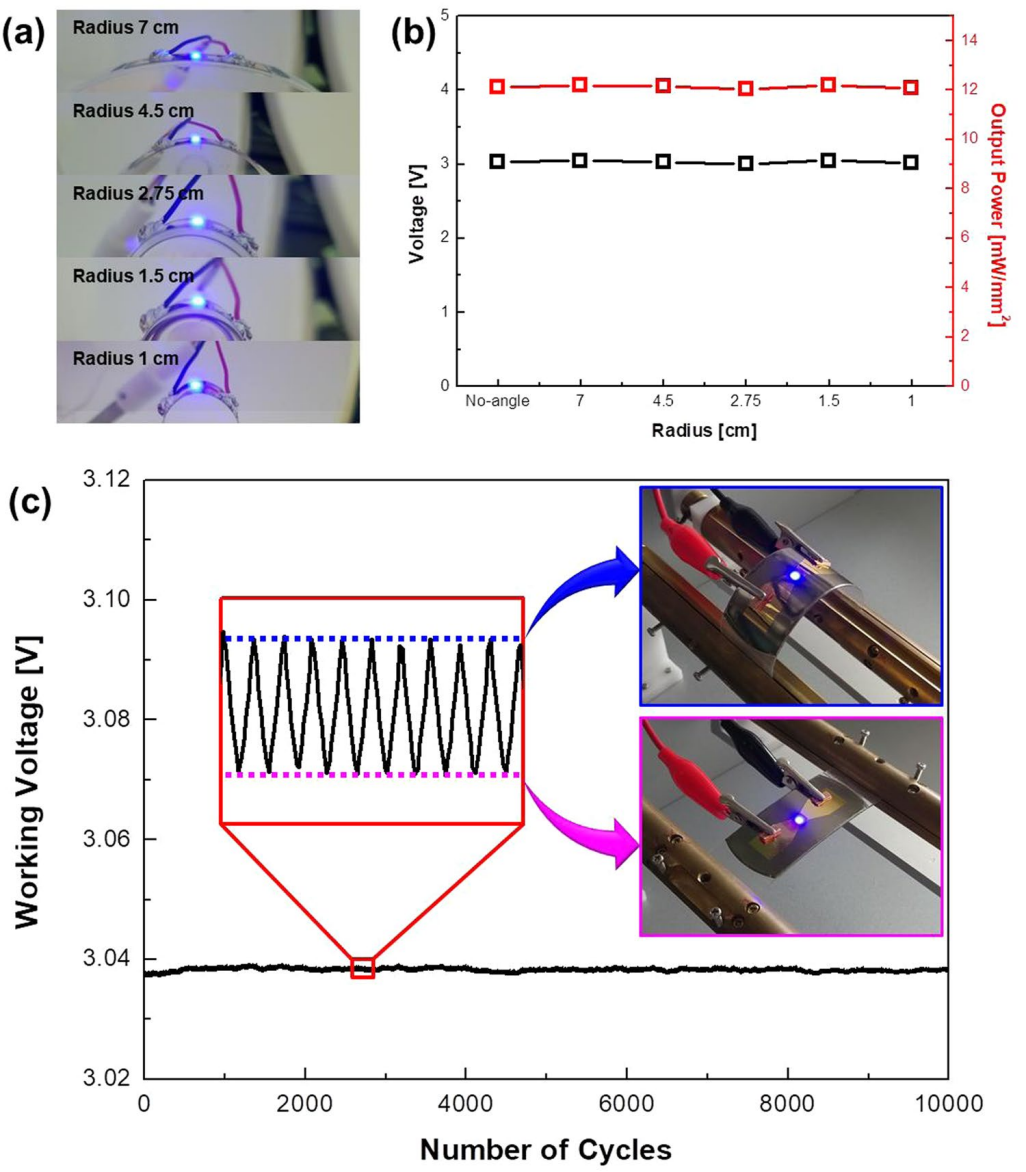
(**a**) Images of light emission of a 10 × 10 μLED array in different bending conditions (radius 7 cm, 4.5 cm, 2.75 cm, 1.5 cm, 1 cm). (**b**) Voltage and output power strain of 10 × 10 μLED array by bending conditions. (**c**) Real-time working voltage change of 10 × 10 μLED array bending test up to 10,000 cycles with bending radius of 2 cm (upper inset: bending state, lower inset: no-bending state).

Figure 4 shows images of light emission of a 10 × 10 μLED array in different bending conditions. The deformable characteristics of 10 × 10 μLED arrays were evaluated in terms of electrical and optical characteristics by decreasing the radius of curvature from 7 cm to 1 cm. The working input current of the 10 × 10 μLED arrays is 4 mA/mm^2^. Even at the minimum bending radius of 1 cm, both the maximum strain of forward voltage and output power density in the 10 × 10 μLED array are 1.2%. To demonstrate the mechanical robustness of the deformable 10 × 10 μLED array, the bending stability was tested under repeated bending events with a bending radius of 2 cm for 10,000 cycles. Figure 4(c) shows the operating voltage of the 10 × 10 μLED array according to the number of bending cycles. The inset graph in Fig. 4(c) shows the working voltage in the bending state and the working voltage in the no-bending state. Because the external tensile stress generated by bending can relax the compressive stress in MQW LED epilayers, reducing the quantum-confined Stark effect (QCSE) by reducing the piezoelectric polarization causes a difference in the working voltage according to bending^27^. The highest working voltage was 3.039 V, and the lowest working voltage was 3.037 V at 4 mA/mm^2^. The standard deviation of the working voltage is 0.0003. It is confirmed that the 10 × 10 μLED arrays have very high reliability in terms of deformability. Finally, considering the similar electrical and optical characteristics of the single chip and the 10 × 10 μLED arrays and the reliability of the deformable characteristics of 10 × 10 μLED arrays, the stamp-imprinting reported in this paper is a stable process that can be repaired.

## Discussion

Stamp-imprinting described in this paper is an accurate and high-throughput technique for mass transfer and repair. The defective chips of the μLED wafer are detected by micro-pulsed laser scanning. Irradiating the desired portion of the UV pulse and adjustment of the irradiation area allow the selected good chips to be picked up, excluding the detected defective chips, and large-area chips can be quickly transferred. These picked-up μLED arrays are imprinted to a functional layer with a controlled adhesion force. The functional layer exposed for 180 s in the first transfer has an adhesion force of 566.2 gf/cm^2^, as well as a sufficient adhesion force of 502.1 gf/cm^2^ compared to the stamp of less than 100 gf/cm^2^ in the second transfer for repair. Therefore, the arrays for repair can be again align-imprinted by selective pick-and-place. The analysis system, pick-and-place, repair technology, and adhesive material described in this paper enable practical and deformable μLED displays with stable optical and electrical characteristics. The performance and durability of the prototype of 10 × 10 μLED arrays were demonstrated under harsh conditions. At a minimum bending radius of 1 cm, the highest strain forward voltage and output power density of 10 × 10 μLED arrays are both 1.2%. The standard deviation of the working voltage is 0.0003 at repeated bending tests with a bending radius of 2 cm for 10,000 cycles. The two-step selective pick-and-place approach proven in this paper can be implemented as an innovative method applicable to various fields such as illumination, sensors, and bio-integrated systems as well as manufacturing deformable defect-free displays.

## Methods

The chip size of the μLEDs in this study was 50 × 50 μm^2^. First, metal-organic chemical vapor deposition (MOCVD) was used to grow u-GaN/n-GaN/MQWs/p-GaN on sapphire substrates. After growth, LED wafers were cleaned in piranha solution. Then, the mesa process was carried out until the n-GaN was exposed for contact between n-GaN and the n-pad. The 0.5-μm LED full structure was etched in BCl_3_/Cl_2_ gas using inductively coupled plasma (ICP). Then, a total thickness of 7 μm GaN was etched under the same conditions as the mesa process to isolate the chip and the chip. The p-spreading layer was formed with indium tin oxide (ITO) by sputtering. The Cr/Au (3000 Å) as the n-pad was deposited using an e-beam evaporator. The Cr/Au (8000 Å) was deposited to form the p-pad. The PDMS stamps for transferring were made by mixing elastomer base and curing agent in a ratio of 10:1 at a curing temperature of 70 °C^28^. The functional layer after coating on the board substrate and then UV exposure for 180 s at a luminance of 7 lm/m^2^ was used for imprinting. Finally, after all the chips were imprinted on the functional layer, it was additionally full-cured for 10 m under the same conditions.

## Supplementary information

Manufacturing method of defect-free prototype 10 × 10 μLED array
