# Supplementary material for: Stable and efficient transfer-printing including repair using a GaN-based microscale light-emitting diode array for deformable displays
Source: Sci Rep. 2019 Aug 9;9:11551. doi: 10.1038/s41598-019-47449-1 (PMC6689111; doi:10.1038/s41598-019-47449-1)
Supplement: Supplementary file 1 — Manufacturing method of defect-free prototype 10 × 10 μLED array [file 41598_2019_47449_MOESM1_ESM.docx]

**Supplementary Information**

**Stable and efficient transfer-printing including repair using a GaN-based microscale light-emitting diode array for deformable displays**

# Jun-Beom Park^1,2^, Keon Hwa Lee^1^, Sang Hoon Han^1^, Tae Jun Chung^1^, Moon Kyu Kwak^3^, Hokyun Rho^2^, Tak Jeong^1*^, and Jun-Seok Ha^2*^

1Korea Photonics Technology Institute, Photonic Device Research Center, 124, Cheomdanventure-ro, Buk-gu, Gwangju, 61007, Republic of Korea

2Chonnam National University, Optoelectronics Convergence Research Center, 77, Yongbong-ro, Buk-gu, Gwangju, 61186, Republic of Korea

*To whom correspondence should be addressed. E-mail: tjeong@kopti.re.kr

# Detailed selective pick-up using UV pulsed excimer and stamp

The selective pick-up of the stamp-imprinting method in this study is an important process including the step of removing defective chips. The μLED chips from the wafer are separated using a UV pulsed excimer (PE) and are picked up as a stamp. Figure S1 shows a schematic diagram related to the more detailed pick-up process than described in the main paper. First, the stamp and μLED wafer were attached by only the sticky property of the PDMS stamp as shown in Figure S1 (a) and (b). The left images of Figure S1 (d) are the sample attached to the stamp and μLED wafer and a cross-section. The front of the chip is well attached to the flat stamp as a whole. After attaching the wafer and the stamp, the chip or chips to be picked up are irradiated using UV PE as shown in Figure S1 (c). The pulse conditions of the UV PE are 40 W power in the size of 60 µm^2^, attenuator 9°, and 50 Hz pulse speed. The UV PE has a wavelength of 248 nm using KrF gas. It has 5 eV depending on the relation between wavelength and energy gap. First, the emitted UV pulse of the UV PE encounters the sapphire (α-Al_2_O_3_) of the μLED wafer. The UV pulse transmits the sapphire because sapphire with a band gap of 8.9 eV can absorb light only with a wavelength lower than 140 nm. Then, when the UV pulse meets μ-GaN, almost all the UV energy is absorbed by μ-GaN, because GaN having a band gap of 3.30 eV absorbs all the light having a wavelength lower than 375 nm. GaN, absorbing energy of the UV pulse, is instantaneously decomposed into Ga(l) and N_2_(g) in a few nanoseconds and is completely separated from the sapphire[1](#_bookmark0),2. Because the chip is attached to the stamp, it is seated on the stamp without movement. This separated chip is like the right images in Figure S1 (d).

The pick-up technique in this study can pick up a large number of chips with one UV pulse. Depending on the area of the UV pulse, more chips can be separated from the sapphire at once. Figures S2 (a) and (b) show the chips picked up according to the UV pulse area. Only the chips in the area of the UV pulse are separated because there is no material that absorbs UV pulses in the isolation region. Figure S2 (c) and (d) are the schematic and process image of scanning a line at 50-Hz speed using pulse picking up a 3 × 3 array at one time. Using this method, it is possible to pick up a large area without inserting another substance or structure in the μLED wafer process.


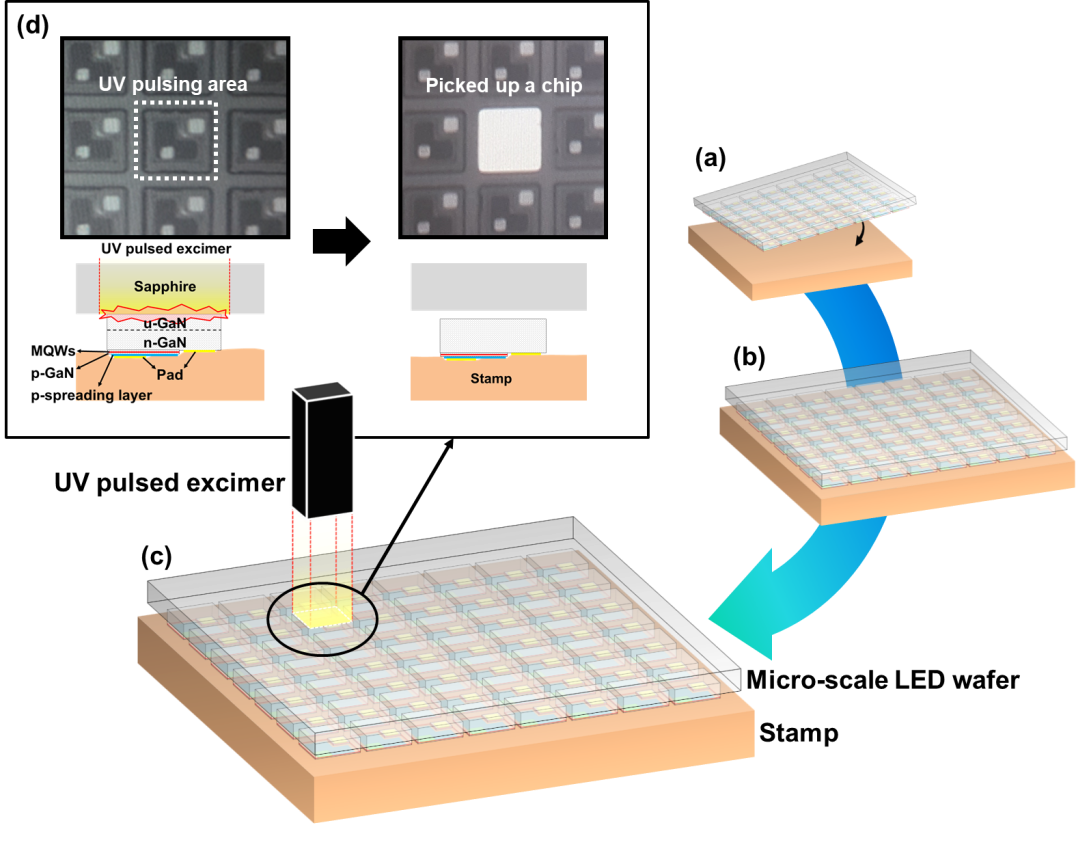


**Figure S1.** Schematic of the pick-up process using UV pulsed excimer and stamp: (a), (b) The μLED wafer is attached to the PDMS stamp by only PDMS adhesive force. (c) Schematic of picking up one chip using UV pulse. (d) Schematic and images of samples picked up using UV pulse after μLED wafers and stamps are evenly attached.


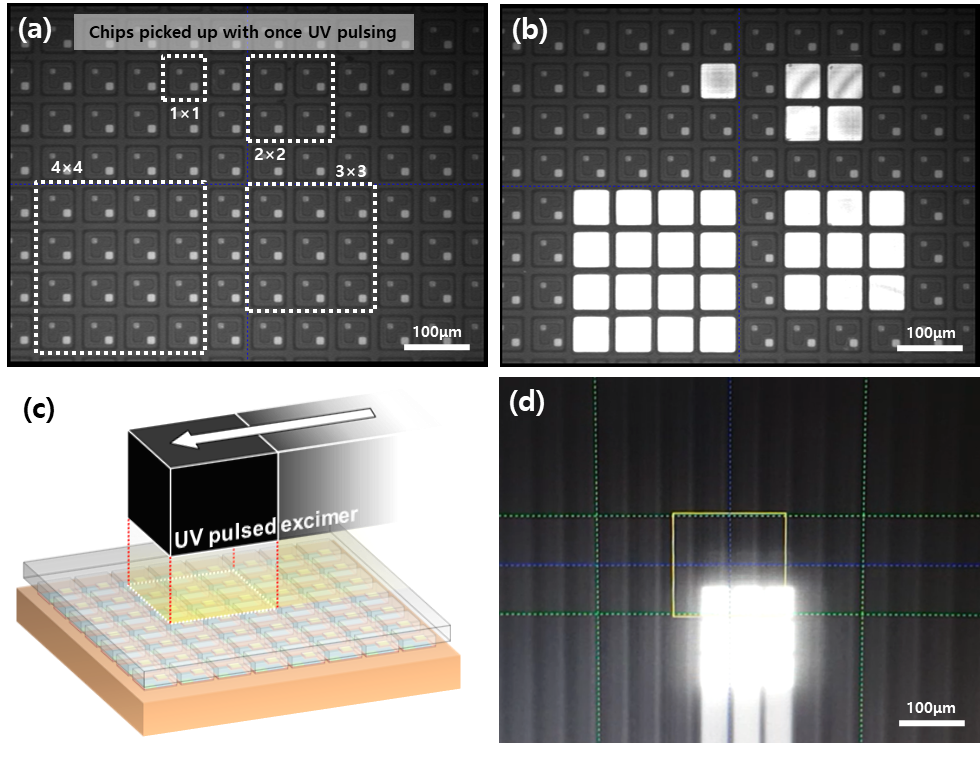


**Figure S2.** (a) Image with μLED wafer and stamp attached. (b) Image of a sample picking up multiple chips at once by adjusting the irradiated area of the UV pulse. (c) Schematic and (d) process image of large-area transfer using a UV pulse capable of picking up a 3 × 3 array at one time.

# Detailed manufacturing method of the prototype 10 × 10 μLED array

We fabricated a monochromatic blue prototype display with 140-μm pitch using PI film as a board substrate. The pitch of the μLED chips on the wafer is 70 μm, but one chip per two chips is picked up to form a 140-μm pitch using simple programming. Figure S3 shows real images and optical microscopy images of the prototype 10 × 10 μLED array fabricated using stamp-imprinting. First, defective chips of the μLED wafers were detected using a pulsed laser emitting at 375-nm wavelength with a diameter of 2 μm. Only good chips, excluding the detected defective chips, were picked up as stamps using UV PE (Figure S3 (a)). In the array picked up by the stamp, the location of the defective chip is empty as shown in Figure S3 (a) as a red dotted outline. The PI board substrate imprinted this array is shown in Figure S3 (b). Good chips are picked up again using UV PE and stamp according to the empty coordinates of the PI board substrate (Figure S3 (c)). This array for repair is aligned-imprinted on the array on the PI board substrate. The PI board substrate after completion of transfer and repair is shown in Figure S3 (d). We have demonstrated that a 10 × 10 μLED array can be fabricated using the stamp-imprinting method represented in this paper, and the possibility of manufacturing μLED displays through this method is demonstrated.


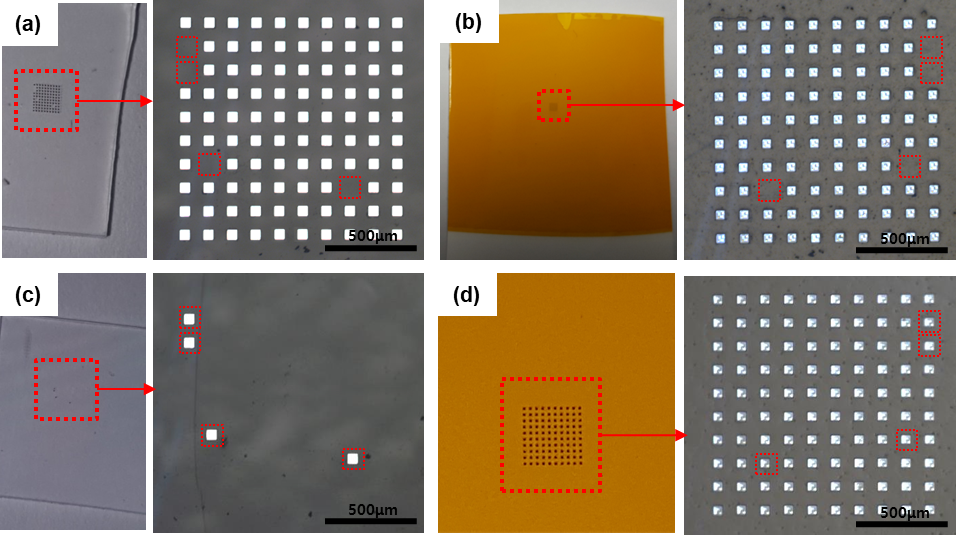


**Figure S3.** (a) Real image and optical microscope image of the sample where only good chips were picked up, excluding the defective chips. (b) Real image and optical microscope image of the array sample of (a) placed on the PI substrate. (c) Real image and optical microscope image of the sample where good chips were picked up for the empty spaces of the array. (d) Real image and optical microscope image of the sample that aligns the array of (c) on the array on a PI substrate.

After finishing transfer, the passivation process is performed to separate the n-electrode and p-electrode using a photosensitive polymer (SU-08). The passivation layer was spin-coated on the PI board substrate to a thickness of 3 μm. It was full-cured in an oven at 180 ℃ for 30 min. The electrodes were connected using ductile Au-based metals. Figure S4 (a) is a schematic of the 10 × 10 array, and Figures S4 (b) and (c) are optical microscope images.


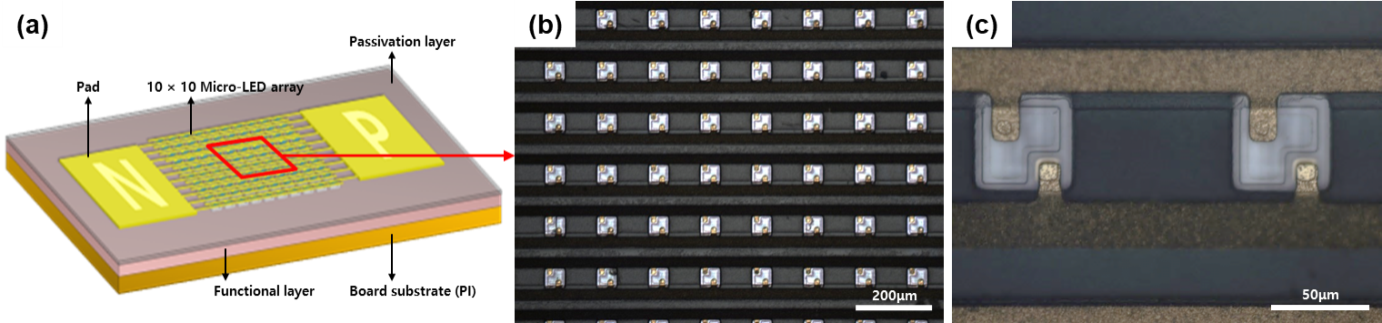


**Figure S4.** (a) Schematic of the 10 × 10 array. (b) Optical microscope image of the 10 × 10 array. (c) Enlarged image of (d).

# References

1. Tetsuzo Ueda, Masahiro Ishida, Masaaki Yuri, *Applied Surface Science*, **216**, 1-4, p.512–518 (2003).
2. W. S. Wong, T. Sands, N. W. Cheung, M. Kneissl, D. P. Bour, P. Mei, L. T. Romano, and N. M. Johnson, *Appl. Phys. Lett.*, **75**, 10, p.1360-1362 (1999).
